# From Aberrant Brainwaves to Altered Plasticity: A Review of QEEG Biomarkers and Neurofeedback in the Neurobiological Landscape of ADHD

**DOI:** 10.3390/cells14171339

**Published:** 2025-08-29

**Authors:** Marta Kopańska, Julia Trojniak

**Affiliations:** 1 Department of Medical Psychology, Faculty of Medicine, University of Rzeszów, 35-959 Rzeszów, Poland; 2 Student Research Club “Reh-Tech”, Faculty of Medicine, University of Rzeszów, 35-959 Rzeszów, Poland; juliatrojniak0@gmail.com

**Keywords:** ADHD, QEEG, quantitative electroencephalography, neurofeedback, NF, biomarker, neural plasticity, neurodevelopmental disorders, neural circuits, Theta/Beta ratio

## Abstract

This critical review synthesizes findings from quantitative electroencephalography (QEEG) to bridge the gap between systems-level neurophysiology and the underlying cellular pathology of Attention-Deficit/Hyperactivity Disorder (ADHD). As a prevalent neurodevelopmental disorder, ADHD diagnosis is challenged by symptomatic heterogeneity, creating an urgent need for objective biological indicators. Analysis of QEEG data reveals consistent neurophysiological patterns in ADHD, primarily an excess of Theta-band activity and a deficit in Beta-band activity. These findings have led to the proposal of specific biomarkers, such as the Theta/Beta Ratio (TBR), and serve as the basis for neurofeedback interventions aimed at modulating brainwave activity. While not a standalone diagnostic tool, this review posits that QEEG-based biomarkers and Neurofeedback responses are systems-level manifestations of putative cellular and synaptic dysfunctions. By outlining these robust macro-scale patterns, this work provides a conceptual framework intended to guide future molecular and cellular research into the fundamental biology of ADHD.

## 1. Introduction

Attention-Deficit/Hyperactivity Disorder (ADHD) is one of the most frequently diagnosed mental health conditions during childhood. Its etiology is notably complex and multifactorial, encompassing an intricate interplay between genetic predispositions and a wide array of environmental factors. These factors include the course of pregnancy, various perinatal influences, and the psychosocial environment throughout a child’s development [1,2]. This profound etiological heterogeneity, compounded by the frequent co-occurrence of ADHD with other neuropsychological disorders, poses a substantial diagnostic challenge for clinicians [3]. Standard diagnostic procedures predominantly rely on clinical interviews and questionnaires completed by parents and teachers. This reliance on subjective reporting underscores the urgent need to identify and validate more objective, biologically based indicators that can effectively support and refine the clinical decision-making process [3]. According to the Diagnostic and Statistical Manual of Mental Disorders, 5th Edition (DSM-5), a diagnosis of ADHD is based on the presence of six or more symptoms of inattention and/or six or more symptoms of hyperactivity-impulsivity. These symptoms must have persisted for at least six months and be inconsistent with the individual’s developmental level. Additionally, symptoms must have been present before the age of 12 and be observable in two or more settings (e.g., at home and at school) [4,5]. The therapeutic management of ADHD is multifaceted, often incorporating pharmacotherapy, behavioral modification techniques, and neurotherapies such as neurofeedback (also known as EEG-Biofeedback) [1,2].

Within this therapeutic landscape, there is a burgeoning interest in electroencephalography (EEG), a non-invasive method that directly measures the brain’s spontaneous neuronal activity. The quantitative analysis of EEG data, a technique known as QEEG (quantitative electroencephalography), facilitates a detailed numerical and visual assessment of brainwave patterns. This allows for the identification of subtle abnormalities in the functioning of specific brain regions, which might otherwise go undetected [6,7]. When compared to other neuroimaging techniques, such as magnetic resonance imaging (MRI) or computed tomography (CT), QEEG offers several distinct advantages. It is a relatively inexpensive procedure, boasts exceptionally high temporal resolution capable of capturing neural events on a millisecond scale, and does not expose the patient to ionizing radiation or powerful magnetic fields [8]. These characteristics, combined with the simplicity of the primary research paradigm—the resting-state recording—make EEG a technology particularly well-suited for routine clinical and screening applications.

This review argues that the translational gap persists because QEEG findings are too often treated as mere clinical correlates. To unlock their full potential, we must reframe these neurophysiological patterns as systems-level manifestations of underlying cellular and molecular dysfunctions. The principal objective of this review is, therefore, to establish a conceptual bridge between the aberrant brainwaves observed in ADHD and their putative synaptic and genetic origins, providing a roadmap for future mechanistic research.

To achieve this, the paper will first critically review the characteristic patterns of bioelectrical activity and key, albeit debated, biomarkers such as the Theta/Beta Ratio (TBR). Subsequently, it will examine the use of QEEG as a basis for neurofeedback interventions—a therapeutic application grounded in neuroplasticity, yet whose efficacy remains a subject of vigorous scientific discussion. Ultimately, by connecting these systems-level phenomena to the search for their underlying biological basis, this review seeks to provide a conceptual roadmap for future research aimed at bridging the gap between brainwaves and the cellular mechanisms of ADHD.

## 2. The Clinical Picture and Etiology of ADHD

A fundamental understanding of the multifaceted nature of Attention-Deficit/Hyperactivity Disorder is essential for the accurate interpretation of its neurobiological correlates and for a realistic evaluation of the potential held by diagnostic tools such as QEEG. ADHD is not a monolithic, homogeneous entity; its clinical presentation and underlying causes are the product of a dynamic and complex interaction among numerous variables.

### 2.1. Symptomatology and Developmental Course

ADHD is clinically characterized by a core triad of symptoms that includes inattention, hyperactivity, and impulsivity [9]. The dimension of inattention manifests as pronounced difficulties in sustaining concentration, completing tasks, and organizing work and daily activities. Individuals exhibiting these symptoms may struggle significantly with attending to details while performing academic assignments or other activities that demand focused mental effort [10,11]. Hyperactivity frequently presents as motor restlessness, fidgeting, or a constant need to change position, making it difficult for the individual to remain seated or still when required [11]. Impulsivity, the third component of the triad, is associated with profound problems with self-control and a propensity for engaging in ill-considered, unpredictable actions without forethought of the consequences.

The clinical manifestation of ADHD undergoes considerable modification throughout an individual’s lifespan. Although the initial symptoms can be challenging to identify with high confidence before the age of four, they typically become most conspicuous and impairing during the early school years [10]. Longitudinal research indicates that the intensity of the core features of ADHD, particularly hyperactivity, tends to diminish with age, becoming markedly less prominent by adulthood [12].

Furthermore, significant gender-based differences exist in the symptomatic expression of the disorder. Statistically, ADHD is diagnosed up to three times more frequently in boys than in girls [13]. Boys more commonly present with externalizing symptoms, such as excessive motor activity, overt impulsivity, and, in some cases, physical aggression. In contrast, girls with ADHD are more likely to exhibit internalizing symptoms. These include pronounced inattention, low self-esteem, and a tendency to either verbalize aggression or direct negative emotions inward. This internalizing symptom profile in girls may be associated with an elevated risk for developing anxiety or depressive disorders later in life [10].

### 2.2. The Multifactorial Etiology of ADHD

The causes of ADHD are highly complex and cannot be attributed to any single, isolated factor. Contemporary scientific understanding posits that the disorder arises from a sophisticated interaction between genetic predispositions and a broad spectrum of environmental influences. The most critical of these risk factors have been systematically compiled and are detailed in Table 1.

The clinical and etiological picture presented here is consistent with the view of ADHD as a neurodevelopmental disorder of a distinctly heterogeneous character. This inherent complexity creates significant challenges in the diagnostic and therapeutic processes, thereby providing a powerful motivation for the search for objective neurobiological markers. The detailed discussion of these markers constitutes the focus of the subsequent sections of this paper [2,10].

## 3. The Neurobiological Basis of ADHD

The array of clinical symptoms and etiological factors associated with ADHD find their direct reflection in specific dysfunctions at the level of the central nervous system. ADHD is not a single pathophysiological entity; it arises from a complex interplay of genetic predispositions and environmental factors that together shape the brain’s development and function. Although the neurobiological underpinnings of ADHD have not yet been fully elucidated, current research points toward a complex web of mechanisms involving genetic architecture, dysregulation of critical neurotransmitter systems, structural and functional alterations in brain networks, and impairments in the fundamental process of neuroplasticity.

### 3.1. Genetic and Environmental Architecture

ADHD is recognized as one of the most heritable psychiatric disorders, with heritability estimates consistently reported to be approximately 76–80% [27,28]. This strong genetic contribution reflects a highly polygenic architecture, where a large number of common genetic variants, each conferring a small effect, collectively increase susceptibility [29]. Genome-wide association studies (GWAS) have begun to identify these variants, implicating genes involved in neurodevelopmental processes, such as FOXP2 [27,29]. Additionally, more recent studies point to the role of KLF (Kruppel-like factors) family genes, which are crucial for proper neuromorphogenesis and have been proposed as candidate genes in ADHD [18,19]. The genetic architecture also includes rare structural mutations of larger effect, known as copy number variants (CNVs), which have been found to overlap with those identified in other neurodevelopmental disorders, including autism spectrum disorder (ASD) and schizophrenia [28,29].

This genetic perspective is further specified by the concept of the heritability of bioelectrical brain activity patterns. Studies in families and twins have demonstrated that certain quantitative electroencephalography (QEEG) parameters, such as the Alpha peak frequency, are heritable traits [30]. This suggests a genetic basis for the fundamental rhythmic organization of the brain’s electrical activity. Moreover, direct links have been identified between specific gene polymorphisms, such as in the COMT gene, and measurable characteristics within the EEG recording [30,31]. These findings support the hypothesis that deviations from normative brain activity may be genetically determined and can be transmitted across generations.

This genetic liability is, however, modulated by a broad spectrum of environmental factors. Prenatal influences, such as maternal smoking and alcohol use, and perinatal factors, like low birth weight, are established risk factors that interact with genetic predispositions. Such gene–environment (GxE) interactions are considered crucial, with evidence suggesting that genetic risk for ADHD is amplified in the presence of environmental adversity [28].

### 3.2. Dysregulation of Neurotransmitter Systems

The genetic and environmental risk factors are thought to converge upon several key neurotransmitter systems, leading to their dysregulation.

#### 3.2.1. Dopaminergic System

The dopaminergic hypothesis remains the most dominant and well-documented theory, positing that ADHD symptoms stem from a deficiency of dopamine in the prefrontal cortex and striatum.

Within this context, particular importance is ascribed to two major dopaminergic pathways:

- The Mesocortical Pathway: This pathway projects to the prefrontal cortex. Its dysfunction is primarily associated with cognitive deficits, problems with attention, and impaired motor control;
- The Mesolimbic Pathway: This pathway projects to the nucleus accumbens. Its aberrant functioning is believed to affect motivation, the experience of pleasure, and the processing of reinforcement and reward, which may underlie the symptom of impulsivity [1,32,33].

This hypothesis is supported by the clinical efficacy of stimulant medications, which act by increasing synaptic dopamine concentrations [34].

Candidate gene studies have provided evidence for the involvement of dopaminergic genes, including dopamine receptors (DRD4, DRD5) and the dopamine transporter (DAT1 or SLC6A3) [1,25,31].

The DRD4 gene is one of the most frequently studied due to a highly polymorphic region in exon 3, containing a variable number of tandem repeats (VNTR) of 48 base pairs [35,36]. Many studies have focused on the 7-repeat (7R) allele. A study in a Turkish population showed an increased transmission of the DRD4 7R allele in children with ADHD, with this association being even stronger in the subgroup of patients who responded well to methylphenidate (MPH) treatment [36]. However, these findings are not consistent across all populations, which highlights significant etiological and ethnic heterogeneity [35,37]. Studies conducted in Irish, Korean, and South Indian populations have not found a significant association between the 7R allele and ADHD [35,37,38]. Furthermore, an association with other alleles has been identified in different ethnic groups:

- The 2-repeat allele in some Chinese populations [35];
- The 6-repeat allele in the Iranian population [39];
- The 6- and 7-repeat alleles in the East Indian population [38].

From a functional standpoint, the 7R allele is believed to mediate a weakened postsynaptic response to dopamine, which is consistent with the hypodopaminergic hypothesis, suggesting a reduced efficiency of signal transmission [36,37].

Similarly, studies on the DRD5 gene have identified an association between ADHD and the 148 bp allele polymorphism (in some studies described as 151 bp) [36,37]. This association also proved to be stronger in patients responsive to MPH in the Turkish trial [36]. However, the functional significance of this DRD5 variant remains unclear, as it is located outside the gene’s coding region [37].

Overall, while these genes are promising candidates, their contribution to ADHD is characterized by a small effect size, with odds ratios typically in the range of 1.5–2.0. This necessitates considering population-specific and clinical subtype differences in research [36,37].

**Figure 1:**
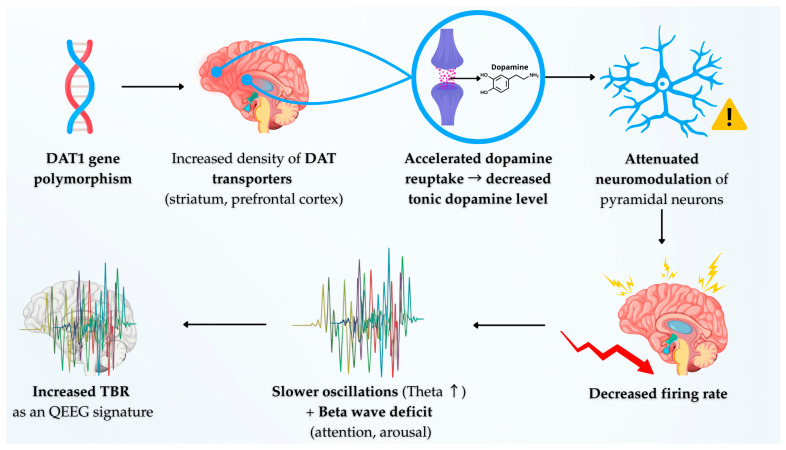
A diagram illustrating the neurobiological cascade in ADHD: from the DAT1 gene polymorphism, through dopaminergic synapse dysfunction, to the electrophysiological signature in the form of an elevated Theta/Beta Ratio (TBR).

The polymorphism of the dopamine transporter gene provides a key example of the gene-protein-EEG nexus. Variants of this gene that lead to an increased density of DAT transporters in the striatum and prefrontal cortex result in an accelerated reuptake of synaptic dopamine [27,40]. At the cellular level, this reduced tonic dopamine level diminishes the neuromodulation of pyramidal neurons in the prefrontal cortex. This may, in turn, result in lower neuronal firing rates and a propensity for slower, less organized oscillations characteristic of the Theta band [28]. Concurrently, a deficit in the Beta band, which is associated with sustained attention, can be viewed as a direct consequence of insufficient dopaminergic stimulation required to maintain a state of high cortical arousal and engagement [41]. Thus, an elevated Theta/Beta Ratio (TBR) can be hypothesized to represent not merely a statistical anomaly, but a direct electrophysiological signature of molecular inefficiency within the dopaminergic system, potentially originating from polymorphisms in the DAT1 gene. This relationship is visually represented in the following Figure 1, which illustrates the full neurobiological cascade in ADHD, from genetic polymorphism to the electrophysiological signature.

#### 3.2.2. Noradrenergic System

The contemporary understanding of ADHD acknowledges a pivotal role for the noradrenergic system. Norepinephrine is fundamental to regulating arousal, vigilance, and executive functions like working memory [34]. The clinical effectiveness of non-stimulant drugs like atomoxetine, which selectively inhibit norepinephrine reuptake, underscores this system’s importance in ADHD pathophysiology [42].

#### 3.2.3. Other Systems

Growing evidence also implicates other neurotransmitter systems. Studies suggest a role for the serotonergic system in impulsivity and emotional regulation, with some association studies showing a link to the serotonin receptor gene HTR1B [40]. The glutamatergic system, the brain’s primary excitatory pathway, is also implicated, with findings of altered glutamate levels in regions like the anterior cingulate cortex [27,43]. Finally, reduced levels of the main inhibitory neurotransmitter, gamma-aminobutyric acid (GABA), have been observed in the somatosensory/motor cortex and striatum of children with ADHD, suggesting an overall imbalance between neuronal excitation and inhibition [27,43,44].

### 3.3. Structural and Functional Brain Alterations

These dysfunctions at the neurochemical level are reflected in observable changes in brain structure and the function of large-scale neural networks.

#### 3.3.1. Structural Findings

Meta- and mega-analyses of structural MRI studies have consistently shown that children with ADHD have, on average, smaller total brain volumes [27,45]. Specifically, reductions have been noted in subcortical regions such as the basal ganglia (caudate, putamen), nucleus accumbens, amygdala, and hippocampus, as well as reduced cortical surface area and thickness, particularly in frontal and parietal regions [27,41]. Furthermore, studies utilizing Diffusion Tensor Imaging (DTI) suggest impaired microstructural integrity of white matter tracts, especially in fronto-striatal and interhemispheric pathways, which may decrease the speed and efficiency of neural communication [27,45]. A key finding from longitudinal studies is a delay in cortical maturation, with the peak of cortical thickness occurring approximately 3 years later in children with ADHD compared to controls, a delay most prominent in the prefrontal cortex [41,45].

#### 3.3.2. Functional Network Dysconnectivity

Functional neuroimaging studies consistently point to dysconnectivity within and between large-scale brain networks. A central finding is the impaired interplay between the Default Mode Network (DMN) and Task-Positive Networks (TPN) [27,44]. The DMN, active during rest and mind-wandering, often shows hyperactivity and insufficient suppression during tasks in individuals with ADHD [44]. Conversely, TPNs, such as the fronto-parietal cognitive control network, often show reduced activation (hypoactivation) during demanding tasks. This dynamic imbalance is thought to underlie core symptoms like attentional lapses and poor task focus. Modulating the interactions between these networks is the Salience Network (SN), which is responsible for switching between internal and external attentional states [27]. Dysfunctions within the SN are believed to contribute to difficulties in the allocation of attention, a central deficit in ADHD.

### 3.4. The Central Role of Impaired Neural Plasticity

A unifying concept that can help explain this constellation of genetic, neurochemical, and network-level dysfunctions is impaired neural plasticity [28]. Neural plasticity refers to the brain’s fundamental ability to change its structure and function in response to experience, forming the basis of learning and development [46]. Many of the top candidate genes implicated in ADHD, such as BDNF and SNAP25, are known to be centrally involved in synaptic transmission and plasticity mechanisms [47]. For instance, genes like SNAP25 are crucial for neurotransmitter release, a prerequisite for the synaptic changes that underlie learning [40,43].

From this perspective, ADHD can be conceptualized not just as a state of neurotransmitter imbalance, but as a disorder of delayed or dysfunctional neuroplasticity, particularly during the sensitive periods of childhood and adolescent brain development [41]. The observed delay in cortical maturation is a direct structural correlate of this altered developmental plasticity [45].

Crucially, this inherent, lifelong capacity for brain modification, or neuroplasticity, while potentially a source of the disorder, also provides the theoretical foundation for non-pharmacological neurotherapeutics [41,46]. Interventions such as Neurofeedback are predicated on this very principle—they aim to leverage the brain’s plastic potential to guide it, through targeted training and operant conditioning, toward more adaptive and efficient functional patterns [46].

Based on this collective evidence, the characteristic alterations in brainwave patterns observed in ADHD can be hypothesized to play a significant role in its pathophysiology, representing the cumulative, functional expression of these multi-level dysfunctions. This provides a strong rationale for the ongoing investigation of QEEG recordings as a source of objective, biologically grounded indicators for this complex disorder, which will be the focus of the subsequent sections.

## 4. QEEG in the Diagnosis of ADHD

**Figure 2:**
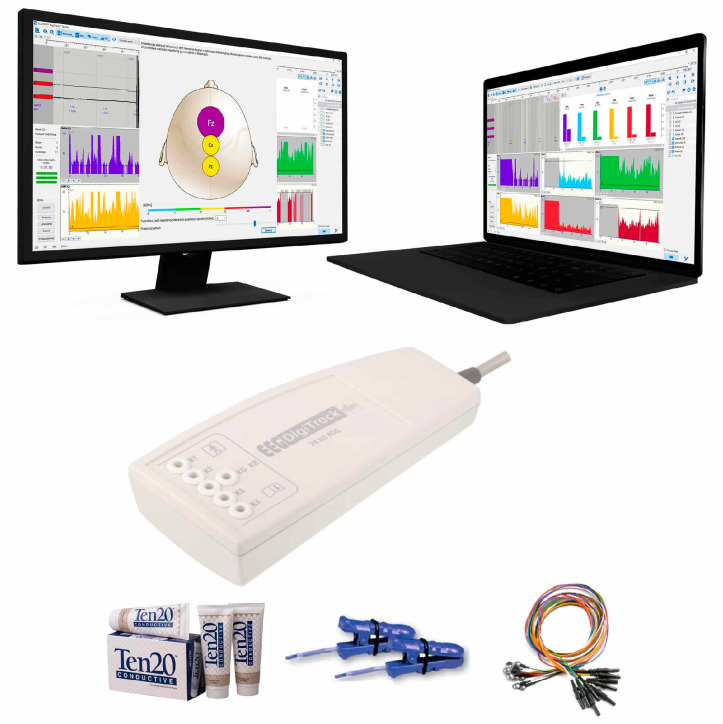
Components of an EEG diagnostic system, including cup electrodes, conductive paste, connecting leads, the DigiTrack EEG headbox and a computer workstation with software for signals acquisition and analysis. Source: (https://koordynacja.com.pl/sklep/neurologia-neurorehabilitacja/eeg-biofeedback/eeg-4-kanalowy/?srsltid=AfmBOooSjuL8kG4qZxuXEYePiZSRO5_4iQ80grmCTedB0YB8YISSM1Zr, accessed on 27 August 2025).

Electroencephalography (EEG) is a non-invasive measurement technique that records the spontaneous electrical activity of the brain via electrodes placed on the scalp. The recorded electrical activity primarily reflects the summation of pre- and post-synaptic potentials that are generated by the simultaneous “firing” of vast populations of neurons [48]. The components of a typical EEG diagnostic system, including the conductive paste, connecting leads, and cup electrodes, are shown in Figure 2.

### 4.1. The Methodology and Potential of QEEG

In stark contrast to structural imaging methods like CT or MRI, or methods that indirectly measure brain metabolism such as fMRI or PET, electroencephalography (EEG) directly reflects the summated postsynaptic activity of millions of neurons with millisecond-level temporal precision [30]. Furthermore, EEG is a relatively inexpensive, entirely non-invasive, and completely safe procedure. It does not expose the patient to any form of ionizing radiation or powerful magnetic fields, and its administration does not necessitate highly specialized or prohibitively expensive equipment [30,49,50]. These attributes, when combined with the simplicity of the resting-state experimental paradigm, render EEG a technology especially predestined for routine, large-scale, and screening applications in clinical settings.

Quantitative electroencephalography (QEEG) represents an advanced, computational extension of classical EEG, which permits a detailed computational analysis of brainwaves [6,7]. Through this technique, the raw EEG signal can be subjected to numerical analysis and subsequently visualized in the form of two-dimensional topographic brain maps. These maps greatly facilitate the precise identification of brain regions exhibiting abnormal or dysregulated activity. Furthermore, modern computational algorithms, such as LORETA (Low-Resolution Brain Electromagnetic Tomography), now enable the three-dimensional localization of the sources of this electrical activity within the brain’s volume, thereby significantly enhancing the spatial accuracy of the examination [30,51].

The capacity of QEEG to detect subtle changes in brainwave amplitudes that correlate with cognitive and behavioral dysfunctions—such as problems with sustained attention, hyperactivity, and impulsivity—makes it a valuable tool in the investigation of ADHD. It is also successfully employed in the diagnosis of a wide range of other neurological and psychiatric disorders [6]. The potential of QEEG is further amplified by the high density of data it acquires and the possibility of applying sophisticated analytical methods, including machine learning algorithms, a potential that has been effectively demonstrated in studies on major depressive disorder, among others [49].

However, despite these promising capabilities, a key and persistent challenge remains: the translation of qualitative research findings into practical, everyday clinical applications. To date, this has not been achieved on a widespread scale [49]. Nevertheless, the utility of QEEG extends beyond being merely a diagnostic tool; it also serves as a foundational element for monitoring treatment progress, for example, in the context of Neurofeedback (EEG-Biofeedback) therapy. This therapeutic modality has been shown to yield particular benefits in populations with ADHD and Autism Spectrum Disorders (ASD) [45], with notable improvements observed in areas such as attention, memory, and the acquisition of relaxation skills [6,7,52].

### 4.2. Characteristic Neurophysiological Patterns in ADHD

A multitude of scientific investigations utilizing QEEG have successfully identified several recurrent patterns of bioelectrical activity that reliably distinguish individuals with ADHD from neurotypical control groups. Although this neurophysiological picture is not entirely uniform across all individuals with the disorder, certain tendencies are particularly well-documented and consistently reported in the scientific literature.

**Figure 3:**
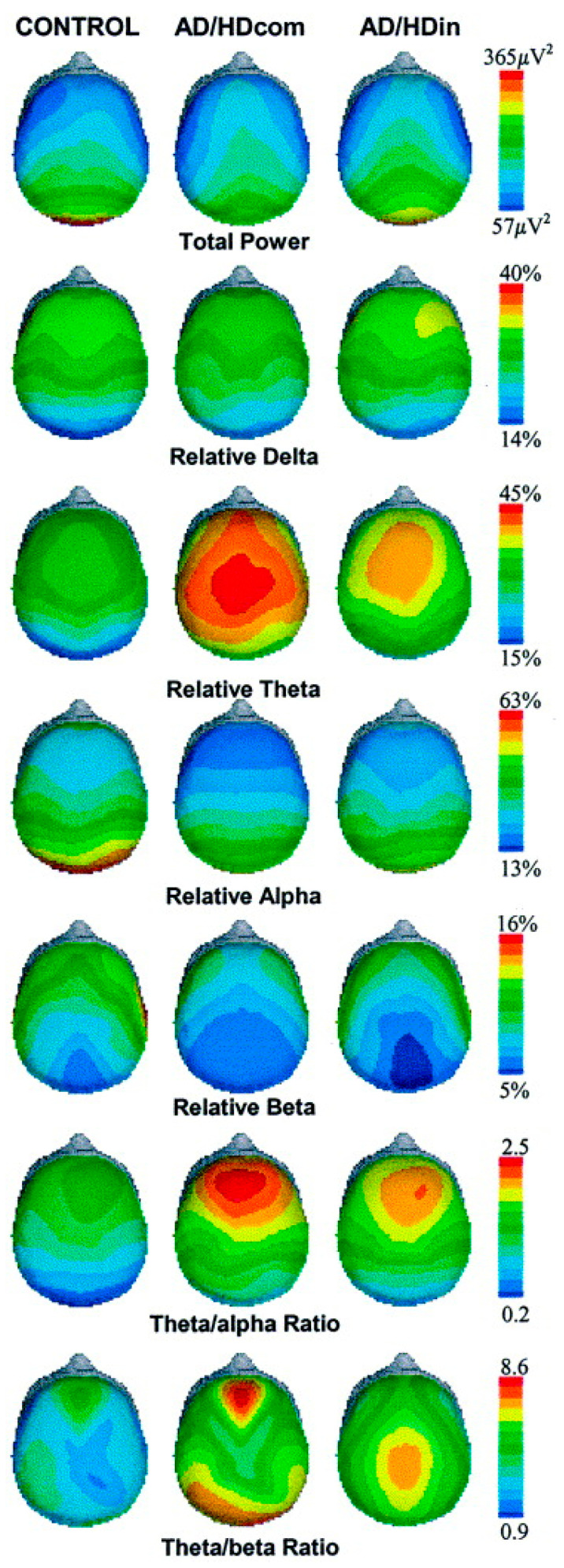
Topographical brain maps illustrating the differences in bioelectrical activity between the control group (**left column**) and ADHD subtypes: combined (**middle column**) and predominantly inattentive (**right column**). Compared to the control group, patients with ADHD, particularly in the combined subtype (AD/HDcom), show a distinct excess of relative Theta power in the frontal regions (indicated in red). This leads to an elevated Theta/Beta ratio, which is considered a key neurophysiological correlate of ADHD symptoms. Figure adapted from Barry et al. (2003) [54].

The most frequently described phenomenon is an excess of slow-wave activity, particularly within the Theta band [53]. This elevated Theta power is most commonly observed in the frontal and central electrode sites, both during resting-state conditions with eyes open and while the individual is engaged in cognitively demanding tasks. Some studies also point to an elevated power in the Delta band [53]. Concurrently, the QEEG recordings of individuals with ADHD often reveal a deficit of fast-wave activity, which manifests as reduced relative power in the Alpha and Beta bands. The differences in bioelectrical activity between the control group and ADHD subtypes are visually represented in the topographical brain maps in Figure 3 [54].

This general neurophysiological profile was confirmed, among others, by Kopańska et al., who observed that children with ADHD exhibited high amplitudes of low-frequency waves (Delta, Theta, and also Alpha) and lower amplitudes of high-frequency waves (including Beta2) relative to the slow waves [55].

It is crucial to emphasize, however, that this “classic” QEEG profile of ADHD can be significantly modified by various factors, most notably the presence of comorbid conditions. A study by Park et al. compared a group with “pure” ADHD, a group with ADHD and a co-occurring Internet Gaming Disorder (IGD), and a healthy control group. The results demonstrated that the “pure” ADHD group was characterized by higher relative Theta power in the frontal regions, a finding consistent with the general model. In contrast, the ADHD + IGD group exhibited lower relative Delta power and higher relative Beta power [53]. The authors of that study suggested that the increase in beta activity in this comorbid group, reflecting heightened cortical activity, might be an effect of repetitive visuospatial processing and the mobilization of executive functions during gaming, leading to an enhancement of neural connectivity.

The combination of the excess Theta and deficit Beta phenomena led to the development of a specific index known as the Theta-to-Beta Ratio (TBR) [53,56]. An elevated TBR is one of the most frequently cited and extensively investigated neurophysiological candidates for a biomarker of ADHD.

These observed patterns are consistently interpreted within the framework of the cortical hypoarousal hypothesis [53,57]. According to this model, the decrease in Beta activity and the concurrent increase in Delta and Theta activity reflect a state of reduced cortical alertness and readiness to process incoming information. Such a neurophysiological state could plausibly underlie the difficulties in sustaining attention, particularly during tasks that demand prolonged cognitive effort. An alternative, complementary concept suggests that these observed patterns may be indicative of a maturational delay of the central nervous system [53].

## 5. Potential QEEG Biomarkers for ADHD

The quest for objective biomarkers in psychiatry represents one of the foremost goals of contemporary medical science. However, the path from laboratory discovery to clinical implementation is fraught with considerable challenges. To date, no biomarkers related to mood disorders in children have been widely integrated into routine clinical practice. This is due, in part, to financial constraints that impede the implementation of appropriate testing, but also due to the profound difficulty in translating complex research data into a tool that allows for genuinely useful clinical decision-making [49]. Furthermore, a significant risk exists for researchers to be misled by creating spurious cause-and-effect chains or overly simplistic mechanistic assumptions [49,58]. It is within this cautious context that one must approach the candidates for QEEG biomarkers in ADHD—with a sense of optimism on one hand, and rigorous scientific circumspection on the other.

Drawing upon the characteristic neurophysiological patterns described previously, scientists are striving to isolate specific, measurable, and replicable indicators—biomarkers—that could objectively support the diagnostic process for ADHD. Although none have yet been universally adopted into clinical practice, two candidates, in particular, have garnered the most significant interest from the scientific community: the Theta-to-Beta Ratio (TBR) and Cross-Frequency Coupling (CFC) [59].

### 5.1. The Theta-to-Beta Ratio (TBR): Promises and Controversies

The ratio of Theta wave power to Beta wave power, known as the TBR index, stands as the longest and most extensively studied candidate for an ADHD biomarker. It is a single-value metric designed to encapsulate the previously described pattern of cortical hypoarousal (an excess of slow waves coupled with a deficit of fast waves). The TBR is typically assessed during a state of quiet rest, with the measurement most often derived from the fronto-central electrode site (Cz) [60,61]. Owing to its conceptual simplicity and its coherence with the dominant hypoarousal theory, an elevated TBR was proposed as a diagnostic marker that could facilitate the diagnosis of ADHD in children. It has been suggested that assessing the ratio of these two frequency bands possesses greater diagnostic sensitivity compared to the independent evaluation of Theta and Beta power alone. 

Despite its popularity and widespread investigation, the diagnostic status of the TBR is the subject of a vigorous and ongoing scientific debate. A significant number of dissenting voices have emerged, challenging both its diagnostic value and its underlying theoretical foundations. The principal objections relate to a purported lack of a significant correlation between the TBR index and objective physiological measures of brain arousal, such as the skin conductance level (SCL). Intriguingly, some researchers have demonstrated that it is a decrease in Alpha wave power, rather than an elevated TBR, that is correlated with an increase in SCL, a direct measure of sympathetic arousal [60,62]. These limitations, combined with the profound heterogeneity of ADHD itself, render the universality of TBR as a reliable marker highly questionable.

However, these inconsistencies do not necessarily invalidate the TBR as a biomarker. Instead, they can be interpreted as compelling evidence for the neurophysiological heterogeneity of ADHD. We propose a conceptual shift away from viewing TBR as a universal indicator for the entire disorder. An elevated TBR may be more accurately conceptualized as a specific biomarker for the subtype characterized by maturational delay and cortical hypoarousal. Conversely, this index would likely be uninformative or even misleading in patients from the ‘hyperarousal’ (excess Beta) or anxiety-related (excess Alpha) subtypes, where attentional deficits stem from different neurophysiological underpinnings. This stratified perspective not only reconciles the disparate findings in the literature but also underscores the critical need for further, more rigorous research to definitively verify the TBR’s clinical utility within specific, biologically defined patient subgroups [60,63].

### 5.2. Cross-Frequency Coupling (CFC): A Network Perspective

A more recent and highly promising candidate for an ADHD biomarker is cross-frequency coupling (CFC), specifically phase-amplitude coupling. CFC is a sophisticated measure that describes the synchronization between neural oscillations occurring at different frequencies, wherein the phase of a slower wave modulates (or controls) the amplitude of a faster wave. It is conceptualized as an indicator that reflects the communication dynamics between large-scale, global neural networks and more localized, specialized neural assemblies. This allows for an assessment of brain dysfunction from a holistic network perspective, rather than focusing solely on isolated brain regions [59].

The most frequently studied form of this phenomenon is the coupling between the phase of Theta waves and the amplitude of Gamma waves [60,64]. It is widely believed that this CFC mechanism plays a pivotal role in a multitude of higher-order cognitive functions, including attentional processes, concentration, and working memory. Research has demonstrated that children with ADHD exhibit a lower degree of Theta-Gamma coupling, particularly during the execution of tasks that demand significant attentional focus, such as mental arithmetic [53,60]. Although the precise mechanisms underlying CFC are not yet fully understood, it represents a fascinating and promising avenue of research that may, in the future, provide more advanced and sensitive biomarkers for the cognitive dysfunctions inherent in ADHD [60].

In summary, while simple, single-value indicators like the TBR have encountered significant limitations, more complex measures that reflect the dynamic interactions of neural networks, such as CFC, are opening new and exciting possibilities. At present, the scientific consensus holds that no QEEG biomarker has achieved a status that would permit its independent use for a definitive clinical diagnosis of ADHD.

## 6. Differentiating ADHD Subtypes Based on QEEG

The profound heterogeneity of ADHD, which is evident in both its clinical presentation and its etiology, is also mirrored at the neurophysiological level. The detailed analysis afforded by QEEG enables the identification of distinct subgroups of patients, each characterized by a unique profile of bioelectrical brain activity. This subtyping approach is significant as it suggests that the single diagnostic label of ‘ADHD’ may encompass several conditions with differing neurobiological underpinnings. This, in turn, implies a critical need for a more differentiated and personalized therapeutic approach.

### 6.1. Classic Subtype Models

Research conducted by Clarke et al. has led to the identification of several key neurophysiological subtypes, which are detailed in Table 2.

### 6.2. Newer Categorization Models

More contemporary analyses, such as those conducted by Byeon et al., have also identified four distinct neurophysiological clusters within the ADHD population. Crucially, this model introduces important methodological considerations regarding the interpretation of QEEG results (Table 3).

### 6.3. Synthesis of Subtype Models

The identification of neurophysiological subtypes of ADHD using QEEG, while conceptually promising, faces significant barriers to its translation into clinical practice due to issues of predictive validation and methodological consistency [71]. Although the existence of heterogeneous bioelectrical profiles challenges a monolithic view of ADHD, their clinical utility remains limited. A key unresolved problem is the low predictive value of QEEG subtypes in the context of treatment response. Despite decades of research, meta-analyses and large-scale studies consistently demonstrate that neither the Theta/Beta Ratio (TBR) nor membership in a specific subtype can reliably predict an individual’s response to stimulant pharmacotherapy or Neurofeedback [72]. Attempts to replicate these associations often yield contradictory results, which, at the current stage of knowledge, precludes the use of QEEG as a biomarker for personalizing therapy. This is further complicated by the lack of evidence for the longitudinal stability of these subtypes, as it is unclear whether a neurophysiological profile diagnosed in childhood persists over time—a prerequisite for its utility as a durable marker [73].

The reliability of QEEG subtyping is additionally undermined by methodological issues. This is starkly exemplified by the identification by Byeon et al. of a patient cluster likely attributable to a measurement artifact [68]. The susceptibility of QEEG results, particularly absolute power metrics, to procedural variables such as electrode impedance highlights the critical need for the standardization of data acquisition and analysis protocols. Without rigorous quality control, the risk of generating clinically misleading profiles remains a significant barrier to the implementation of QEEG in ADHD diagnostics.

However, rather than viewing these challenges as an invalidation of the subtyping approach, a critical analysis suggests they can be better understood by integrating existing models into a more cautious, speculative framework. We therefore propose a heuristic conceptualization that, instead of treating subtypes as mutually exclusive, synthesizes the findings of researchers like Clarke et al. and Byeon et al. into three potential dimensions of ADHD heterogeneity.

The first proposed dimension, a Maturational/Structural Deficit, could integrate the ‘Maturational Lag’ model with ‘Group A’ (elevated Delta power) [65,67,68]. The neurobiological underpinnings for such a dimension might be the structural brain changes seen in MRI studies, such as delayed cortical maturation, which act as physical correlates of CNS immaturity. The second speculative dimension, a Thalamocortical Dysrhythmia, could combine the classic ‘Hypoarousal Type’ (excess Theta, elevated TBR) with ‘Group C’ (elevated Theta power) [65,67,68]. This profile may reflect the fundamental dysfunctions within dopaminergic and noradrenergic systems that are central to ADHD pathophysiology. Finally, a third dimension could be an Internalizing/Anxious Profile, synthesizing the ‘Excess Alpha Waves Subtype’ with ‘Group B’ (elevated slow Alpha power) [65,67,68]. It is plausible that this profile does not represent ‘pure’ ADHD, but rather a phenotype where inattentive symptoms are secondary to co-occurring anxiety or affective disorders.

This proposed framework is not presented as a definitive model but as a potential tool to reconcile contradictory data. Viewing heterogeneity through these dimensions, rather than rigid categories, may provide a more nuanced basis for patient stratification in future validation studies.

## 7. The Application of QEEG in Neurofeedback Therapy

Beyond its diagnostic applications, quantitative electroencephalography (QEEG) provides the fundamental basis for innovative therapeutic methods aimed at the direct modification of abnormal brain activity patterns. The most prominent of these in the context of ADHD is Neurofeedback (also known as EEG-Biofeedback), a technique that enables a patient to consciously learn the self-regulation of their central nervous system functions [74,75].

### 7.1. The Principle of Neurofeedback

Neurofeedback is a sophisticated form of training grounded in the principles of operant conditioning. The entire process is built upon a real-time feedback loop:

1. Measurement: The patient’s bioelectrical brain activity is continuously measured using EEG;
2. Processing: Specialized software analyzes the incoming EEG signal in real-time and extracts key parameters, such as the amplitude of specific brainwaves in a particular area of the brain;
3. Feedback: The processed information is then presented back to the patient in an accessible and engaging format, typically visual or auditory. This often takes the form of a video game, where progress and success depend on the patient’s ability to maintain the desired state of brain activity;
4. Learning Self-Regulation: The theoretical goal of this process is the consolidation of new, more adaptive patterns of brain functioning [76,77,78,79,80,81,82,83].

The overarching goal of the therapy is to reinforce desired brainwaves (e.g., those associated with focus) and to suppress or inhibit undesired brainwaves (e.g., those associated with mind-wandering), all in accordance with the patient’s unique, individual QEEG profile [84,85].

### 7.2. Brainwaves and Training Goals in ADHD Therapy

Effective neurofeedback therapy hinges on a clear understanding of the functions of the various brainwave frequency bands and their typical abnormalities in ADHD [74]. The primary frequency bands of interest include: Delta (0.5–4 Hz), Theta (4–8 Hz), Alpha (8–12 Hz), Beta (>12 Hz), SMR (12–15 Hz), Beta1 (15–18 Hz), Beta2 (>18 Hz), and Gamma (>30 Hz). In a healthy, functioning brain, these waves are produced simultaneously, each with a specific amplitude and frequency, and normative ranges for these parameters are indicative of proper brain function [86] (Table 4).

In conclusion, Neurofeedback is a complementary therapeutic method that, in a targeted manner, stimulates specific brain regions and rhythms to improve their overall functioning. Particular benefits are observed in the enhancement of sustained attention, memory, and the acquisition of relaxation skills, making it a highly promising therapeutic option for individuals with ADHD.

### 7.3. The Efficacy, Controversies, and Limitations of Neurofeedback in ADHD

Although the goal of Neurofeedback therapy is for an individual to consciously learn the self-regulation of central nervous system functions, and its theoretical premise is the consolidation of new, more adaptive patterns of brain functioning, the extent to which these acquired skills transfer to daily functioning and lead to durable clinical effects remains a subject of intense scientific debate. Numerous studies point to the significant influence of the placebo effect and other non-specific factors [93,94,95].

One of the fundamental challenges in research on neurofeedback efficacy is the difficulty in designing a credible, methodologically sound control group. In an ideal research model (a Randomized Controlled Trial or RCT), a “sham-neurofeedback” condition is employed, wherein the patient performs an identical task (e.g., playing a video game), yet the feedback presented is non-contingent and has no real correlation with their brain’s bioelectrical activity [96]. Many published studies, particularly earlier ones, lacked an adequately designed and blinded control group, which significantly complicates the disentanglement of the specific effects of EEG training from the influence of non-specific factors [97].

This leads to a crucial question regarding the nature of the observed changes: does the improvement in a child’s functioning stem directly from the normalization of brainwaves, or is it an effect of the mere fact that the child is regularly and intensively training their concentration in front of a computer under a therapist’s guidance? Factors such as motivation, the systematic nature of the training, and a positive therapeutic relationship can, in themselves, have a therapeutic effect, independent of the specifics of the EEG training [98].

Findings from meta-analyses, such as the one by Arns et al., often underscore this complexity. They indicate that while assessments by unblinded observers (e.g., parents or teachers) often report significant improvements in symptoms of inattention and hyperactivity, the results from objective neuropsychological tests are frequently less conclusive [74]. This discrepancy between subjective and objective outcome measures forms the crux of the debate on the true efficacy of Neurofeedback as a standalone intervention for ADHD.

Beyond the debate over clinical efficacy, a more fundamental controversy surrounds the actual mechanism of action of Neurofeedback. This ‘mechanistic gap’ raises a critical question: Do clinical improvements stem from the lasting normalization of brainwave patterns, or from non-specific cognitive training effects? Crucially, the evidence that successful neurofeedback leads to durable changes in the resting-state QEEG profile is inconsistent. This lack of a clear link between the purported mechanism (EEG normalization) and the outcome (symptom reduction) suggests that therapeutic benefits may be driven more by the structured training of executive functions, rather than the Biofeedback component itself.

### 7.4. A Review of Clinical Studies on the Efficacy of Neurofeedback

Although neurofeedback has been used for several decades, its status as an evidence-based intervention for treating ADHD remains uncertain. A key challenge is conducting rigorous, well-controlled clinical trials that can distinguish the specific effects of EEG training from non-specific factors, such as the placebo effect, regular contact with a therapist, or patient motivation.

Table 5 below presents a summary of selected, significant clinical trials registered in the ClinicalTrials.gov database. The focus is on key aspects such as the study population (children and adults), the type of NFB intervention applied, the nature of the control group, and the primary measures of efficacy. An analysis of these trials reveals methodological diversity—from Slow Cortical Potential (SCP) training, through standard frequency training (e.g., Beta/Theta), to innovative mobile solutions. A crucial element of many newer studies is the use of a double-blind design with a control group receiving sham neurofeedback, which significantly enhances the reliability of the results. The trials encompass the assessment of core ADHD symptoms (inattention, hyperactivity), as well as executive functions and the overall functioning of patients.

The efficacy of neurofeedback in treating ADHD is a subject of intense scientific debate, which is reflected in the results of completed clinical trials. Early studies provided promising data; however, newer, methodologically more rigorous studies question the specificity of this intervention.

In one of the more frequently cited studies, Gevensleben et al. (NCT00723684) compared neurofeedback with computer-based cognitive skills training. However, EEG neurofeedback did not prove to be better than placebo neurofeedback in alleviating ADHD symptoms in children with ADHD [99].

Subsequent large-scale, multi-center studies yielded similar conclusions. The multi-center “ESCA-Studie” (NCT01841151) did not show that neurofeedback, in two different protocols, was more effective than an active control group, which consisted of working memory training. All groups showed similar improvement, suggesting that the observed effect might be due to non-specific factors such as motivation or regular contact with a therapist [100]. Similar conclusions emerged from a study on an adult population (NCT01883765), where neurofeedback was not found to be superior to metacognitive training and group cognitive-behavioral therapy [101].

The biggest challenge for proponents of the method, however, comes from a study published by the Neurofeedback Collaborative Group (NCT02251743). This was a large, rigorous double-blind study with a control group receiving sham neurofeedback (placebo). It found no statistically significant difference between real neurofeedback and placebo. Both groups achieved similar, sustained improvement, which strongly suggests that the clinical benefits may be attributed to the placebo effect and other non-specific factors. These results constitute some of the strongest evidence challenging the specific effectiveness of the tested NFB protocol [102].

In conclusion, the most rigorous evidence from recent years does not confirm that the tested NFB protocols have a specific effect that surpasses the placebo effect or other active interventions. The contemporary scientific discussion focuses on the role of non-specific factors in the observed therapeutic effects and on the search for new protocols or patient subgroups for whom this form of therapy might indeed prove effective.

## 8. Discussion, Synthesis, and Future Directions

**Figure 4:**
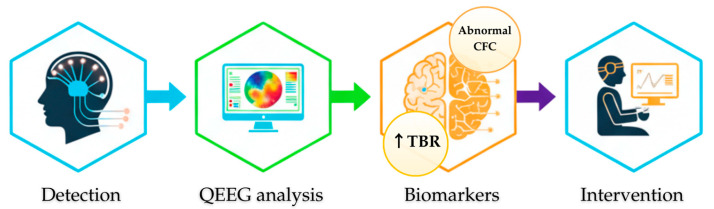
A diagram illustrating the role of QEEG in the ADHD diagnostic and therapeutic process. The process includes EEG signal detection, its quantitative analysis (QEEG) to identify neurophysiological biomarkers (e.g., TBR), which serves as the basis for planning and monitoring therapeutic interventions, such as Neurofeedback.

This review summarizes the role of quantitative electroencephalography (QEEG) in ADHD research, from its characteristic neurophysiological patterns to its application in Neurofeedback therapy. As has been demonstrated, the most frequently reported pattern in the QEEG of individuals with ADHD is an excess of slow Theta waves with a concurrent deficit of fast Beta waves. This phenomenon, interpreted as an expression of cortical hypoarousal, became the basis for developing the Theta/Beta Ratio (TBR) index as a potential biomarker. The data analysis also confirms that ADHD is not a monolithic entity, which is reflected in the existence of distinct neurophysiological subtypes that may require differentiated therapeutic approaches [55]. The overall role of QEEG in the diagnostic and therapeutic process for ADHD is summarized in the diagram below—Figure 4.

However, to fully realize the potential of these findings, it is necessary to move beyond the descriptive level and create a bridge to the underlying cellular mechanisms. Acknowledging this complexity, we propose a series of targeted, heuristic hypotheses designed to serve as starting points for untangling these intricate relationships. It is crucial to emphasize, however, that fundamental challenge in bridging neurophysiology with molecular biology is the ‘many-to-one problem’, which posits that a single macroscopic observation can arise from multiple, distinct underlying mechanisms. Consequently, a characteristic electrophysiological signature like an elevated Theta/Beta Ratio (TBR), while a robust finding, should not be viewed as a unique marker of a single pathology. Instead, it may represent a ‘final common pathway’ for various pathophysiological processes. For instance, while consistent with the hypothesis of dopaminergic dysfunction, an elevated TBR could theoretically also stem from a primary dysregulation of the noradrenergic system, an imbalance in GABA/glutamate signaling, or subtle alterations in thalamocortical architecture. The causal links proposed in this review must therefore be interpreted with appropriate caution: they are not presented as definitive pathways but as heuristic models intended to delineate the most promising avenues for future research. Disentangling these potential contributors will be a critical task for subsequent studies, necessitating a multi-modal approach that integrates QEEG with genomics, proteomics, and advanced structural neuroimaging.

Hypothesis 1: The Cellular Basis of the Theta/Beta Ratio (TBR). We propose that an elevated TBR is a direct electrophysiological signal of dysfunction at the level of neural circuits, driven by specific cellular changes. The excess of frontal Theta power may result from reduced tonic activity of dopaminergic neurons in the mesocortical pathway, leading to a state of insufficient prefrontal cortex arousal. Concurrently, the deficit in Beta power, which is key for sustained attention, may reflect a disturbed balance between excitation (glutamatergic) and inhibition (GABAergic) in thalamo-cortical loops, which precludes the maintenance of a stable state of cognitive engagement [27].

Hypothesis 2: Synaptic Inefficiency as the Source of Deficits in Cross-Frequency Coupling (CFC) [59]. The reduced phase-amplitude coupling between Theta and Gamma waves (Theta-Gamma coupling), observed in patients with ADHD during cognitive tasks, suggests a defect in neuroplasticity mechanisms [60]. We hypothesize that its source is an inefficiency of the presynaptic machinery. Polymorphisms in genes crucial for plasticity, such as SNAP25 or BDNF, may impair the precise, time-dependent coordination of neurotransmitter release, which is necessary for the nesting of fast Gamma oscillations (reflecting local processing) within the slower, global Theta rhythm (serving as a temporal framework for memory processes) [40,43,47,64,77].

The formulation of precise, empirically verifiable hypotheses, such as those linking specific oscillatory dysfunctions to the genetics of synaptic machinery, constitutes a critical translational step. This approach reframes the utility of quantitative electroencephalography (QEEG), elevating it from a correlational diagnostic tool to a scientific platform for generating targeted research questions for basic neuroscience. This transition—from observing systems-level neurophysiological phenomena to probing their putative cellular and molecular underpinnings—is essential for a mechanistic understanding and for ultimately elucidating the fundamental biology of ADHD.

## 9. Conclusions

This review provides a critical synthesis of research on quantitative electroencephalography (QEEG) in ADHD, positing that characteristic neurophysiological signatures, such as the elevated Theta/Beta Ratio (TBR), extend beyond the role of epiphenomenal clinical correlates. It is postulated that they represent, rather, a systemic, measurable manifestation of their underlying pathophysiological substrates, encompassing aberrations in the dynamics of the dopaminergic system and the impairment of fundamental processes of synaptic plasticity. Importantly, QEEG data also provide empirical evidence for the significant heterogeneity of ADHD, revealing the existence of distinct bioelectrical subtypes, which implies the necessity of patient stratification and the personalization of interventions. A translational implication of this paradigm is Neurofeedback therapy, which, by leveraging endogenous mechanisms of neuroplasticity, represents an attempt at the targeted modulation of the identified dysfunctional neuronal oscillations. The results of clinical trials from recent years, however, do not confirm that the tested NFB protocols have a specific effect that surpasses the placebo effect or other active interventions. In conclusion, this work argues that the multi-scale integration of data—from genetics and cell biology to macroscopic QEEG indicators—is a prerequisite for overcoming the explanatory gap in the etiopathogenesis of ADHD. Such a perspective offers a viable path toward the development of biologically grounded stratification and predictive biomarkers, paving the way for precision medicine in the diagnosis and treatment of this complex neurodevelopmental disorder.

## Figures and Tables

**Table 1:** Principal risk factors associated with the occurrence of ADHD.

Category of Factors	Example Risk Factors	Source
Genetic Factors	Functional variants or combinations of multiple genes (no single “ADHD gene” exists)	[1,14]
	The dopamine transporter gene (DAT1) and the dopamine D4 receptor gene (DRD4)	[15]
Epigenetic Factors	Differences in DNA methylation profiles in DRD4 and DAT1, linked to childhood ADHD symptoms.	[16,17]
	DNA methylation in KLF13	[18,19]
	Epigenetic changes in the DRD4 and DRD5 genes	[16,17]
Prenatal and Perinatal Factors	Preterm birth	[20]
	Low or very low birth weight	
	Preeclampsia during pregnancy	[10,21]
	Maternal hypertension	
	Maternal overweight or obesity	[22]
	Maternal exposure to smoking and illicit drugs during pregnancy	[23,24]
	Perinatal hypoxia	[25,26]
	Postnatal inflammation in the infant	[25]
Environmental and Family Factors	Negative parental reactions and harsh disciplinary practices	[2]
	Excessive exposure to media and screen time	[2]

**Table 2:** Neurophysiological subtypes of ADHD according to the model by Clarke et al. [60,65,66,67].

Subtype Name	Key QEEG Characteristics	Clinical Interpretation
Maturational Lag Type	An excess of slow Theta waves and a deficit of fast Beta waves	This pattern is considered evidence of slower brain development and immaturity of the central nervous system compared to neurotypical peers
Thalamocortical Dysrhythmia (“Hypoarousal”) Type	Elevated power in both Theta and Beta bands, which translates to an increased TBR	This pattern is sometimes linked to dysfunction in the thalamocortical loop and may also be present in other psychiatric disorders, making it non-specific to ADHD
“Hyperarousal” Type	Characterized by over-activity and increased power of Beta waves	This profile may be clinically associated with a tendency toward aggressive outbursts, anger, and general irritability
Excess Alpha Waves Subtype	Increased power of Alpha waves	This profile is strongly associated with the co-occurrence of internalizing disorders, such as anxiety, depression, or emotional dysregulation. It is thought to reflect the presence of a comorbid condition rather than “pure” ADHD

**Table 3:** Neurophysiological subtypes of ADHD according to the model by Byeon et al. [68,69,70].

Subtype Name	Key QEEG Characteristics	Clinical Interpretation
Group A	Elevated relative Delta power with lower Theta activity	Consistent with the maturational lag type; this has been corroborated by MRI studies indicating smaller cortico-striatal regions
Group B	Elevated relative power of slow Alpha waves	Alpha waves are associated with arousal; their elevated power correlates with symptoms of depression and anxiety, which can mimic the symptoms of ADHD
Group C	Elevated Theta power with insufficient Alpha power	Consistent with the classic “hypoarousal” type
Group D	Increased absolute power of fast Alpha and Beta waves	This is likely a measurement artifact. An excessive or insufficient amount of electrode paste can distort the measurement of absolute power. The relative power in this group was within the normal range

**Table 4:** The main brainwave frequency bands and their role in Neurofeedback therapy for ADHD.

Wave	Range [Hz]	Function	ADHD Manifestation	Training Goal	Source
Alpha	8–12	The Alpha rhythm dominates in a state of relaxed wakefulness with eyes closed, but is also linked to focus and creativity. Increasing its amplitude can lower anxiety levels	Although a deficit of alpha waves is often observed, in subtypes with comorbid anxiety, its power may be elevated	Dependent on the patient’s profile; may involve reinforcing alpha to improve relaxation capabilities and reduce anxiety	[87,88,89]
Theta	4–8	This rhythm is associated with memory processes and association, but also with states of dreaminess and reduced concentration	An excess of theta waves in the frontal lobes is a key correlate of problems with concentration and attention	The primary goal in many ADHD protocols is to inhibit (decrease) the amplitude of Theta waves	[90]
Beta	>12	Beta waves are linked to alertness, concentration, logical thinking, and the processing of external stimuli. A specific sub-band, the SMR rhythm, is associated with calm focus and motor control	A deficit of Beta waves is often observed, particularly in the SMR band	A fundamental element of therapy is to reinforce (increase) the amplitude of SMR and/or Beta1 (15–18 Hz) waves. This training aims to improve concentration and reduce hyperactivity and impulsivity	[83,84,85,91,92]
SMR	12–15				
Delta	0.5–4	Delta is the rhythm of deep, restorative sleep; its presence during wakefulness is indicative of pathology	N/A ^1^	Not typically a direct training target in standard protocols	[84,85]
Gamma	>30 Hz	Gamma is associated with higher cognitive processes and the integration of information	N/A		

^1^ N/A—not applicable.

**Table 5:** Key Characteristics of Selected Clinical Trials on Neurofeedback as a Therapeutic Intervention for ADHD.

NCT ID	Study Title	Enrollment	Interventions	Primary Outcome Measure(s)	Study Status	Age	Sex	Study URL
NCT00723684	Efficacy of EEG Neurofeedback in the Treatment of Children with ADHD	63	Placebo EEG Neurofeedback; EEG-Neurofeedback	Change in ADHD-IV Rating Scale	Completed	8 to 12 years	All	https://clinicaltrials.gov/study/NCT00723684, accessed on 16 June 2025
NCT00886483	Neurofeedback Treatment for Children with ADHD	39	Active neurofeedback; Sham neurofeedback	Change in the ADHD Rating Scale (ADHD-RS)	Completed	7 to 11 years	All	https://clinicaltrials.gov/study/NCT00886483, accessed on 16 June 2025
NCT01692548	Neurofeedback Training in Children with ADHD	36	Neurofeedback	Improvement in inattention and hyperactivity/impulsivity symptoms as measured by the FBB-HKS questionnaire	Completed	7 to 9 years	All	https://clinicaltrials.gov/study/NCT01692548, accessed on 16 June 2025
NCT01841151	Neurofeedback in the Treatment of ADHD in Children and Adolescents	202	SCP training; Live Z-score training; WM training	Change in the parent-rated ADHD symptoms scale (FBB-ADHD)	Completed	7 to 13 years	All	https://clinicaltrials.gov/study/NCT01841151, accessed on 16 June 2025
NCT01879644	Combined Treatment (Neurofeedback and Stimulants) in ADHD	120	Neurofeedback; Methylphenidate; Parent Education; Standard Treatment	Change in teacher ratings of ADHD symptoms	Active, Not Recruiting	7 to 10 years	All	https://clinicaltrials.gov/study/NCT01879644, accessed on 16 June 2025
NCT01883765	Neurofeedback and Metacognitive Training in Adults with ADHD	118	Active neurofeedback; Sham neurofeedback; Metacognitive Training	Change in Conners’ Adult ADHD Rating Scale (CAARS-O)	Completed	18 to 50 years	All	https://clinicaltrials.gov/study/NCT01883765, accessed on 16 June 2025
NCT02251743	A Randomized Controlled Trial of Neurofeedback for ADHD	144	Neurofeedback treatment	Change in the ADHD Rating Scale—Parent Version	Completed	7 to 10 years	All	https://clinicaltrials.gov/study/NCT02251743, accessed on 16 June 2025
NCT02358941	A Comparison of Neurofeedback and Computerized Cognitive Training	102	Neurofeedback training; Computerized cognitive training	Change in the Swanson, Nolan, and Pelham (SNAP-IV) scale	Completed	7 to 12 years	All	https://clinicaltrials.gov/study/NCT02358941, accessed on 16 June 2025
NCT02572180	Combined Biofeedback and Neurofeedback Training in ADHD	90	EMG-based biofeedback training; NIRS-based neurofeedback training	Change in ADHD Rating Scale IV (ADHD-RS-IV)	Unknown	7 to 15 years	All	https://clinicaltrials.gov/study/NCT02572180, accessed on 16 June 2025
NCT02754336	Comparison of Working Memory Training with Neurofeedback in Children with ADHD	6	Cogmed Robomemo, working memory training; Othmer, neurofeedback	Change in working memory test scores	Unknown	8 to 12 years	All	https://clinicaltrials.gov/study/NCT02754336, accessed on 16 June 2025
NCT02778360	Neurofeedback as an Adjunctive Treatment for ADHD	179	Neurofeedback NFT; Methylphenidate MPH	Change in ADHD-RS-IV (Parent Version)	Recruiting	6 to 12 years	All	https://clinicaltrials.gov/study/NCT02778360, accessed on 16 June 2025
NCT04112082	Efficacy of a Mobile Neurofeedback System in Adults with ADHD	70	Mobile neurofeedback; Treatment as usual	Change in the Adult ADHD Self-Report Scale (ASRS-SK)	Recruiting	18 to 60 years	All	https://clinicaltrials.gov/study/NCT04112082, accessed on 16 June 2025
NCT04408521	Efficacy of EEG Neurofeedback for the Treatment of Adult ADHD	5	NEUROFEEDBACK; CONTROL	Change in clinician rating of ADHD symptoms	Unknown	18 to 50 years	All	https://clinicaltrials.gov/study/NCT04408521, accessed on 16 June 2025
NCT04469335	Mobile Neurofeedback in Children with ADHD (m-NFB-ADHD)	165	Mobile neurofeedback; Sham mobile neurofeedback; Medication + mobile neurofeedback; Medication + sham mobile neurofeedback	Change in the parent-rated ADHD scale (FBB-HKS)	Unknown	7 to 12 years	All	https://clinicaltrials.gov/study/NCT04469335, accessed on 16 June 2025
NCT05635318	Quantitative EEG Neurofeedback as an Add-on Therapy for ADHD	102	FDA-approved medications for ADHD plus Quantitative EEG Neurofeedback; FDA-approved medications for ADHD	Change in the ADHD-RS-V rating scale	Unknown	6 to 12 years	All	https://clinicaltrials.gov/study/NCT05635318, accessed on 16 June 2025
NCT06142786	Individualized Alpha Frequency-based Neurofeedback in ADHD	60	Enhancement of individualized upper alpha band and suppression of lower alpha band; Sham neurofeedback	Change in the Swanson, Nolan and Pelham (SNAP) scale	Unknown	8 to 12 years	All	https://clinicaltrials.gov/study/NCT06142786, accessed on 16 June 2025

## Abbreviations

The following abbreviations are used in this manuscript:

ADHD	Attention-Deficit/Hyperactivity Disorder
ASD	Autism Spectrum Disorders
CFC	Cross-Frequency Coupling
CNV	Copy Number Variant
COMT	Catechol-O-Methyltransferase
CT	Computed Tomography
DAT1	Dopamine Transporter 1
DMN	Default Mode Network
DRD4	Dopamine Receptor D4
DRD5	Dopamine Receptor D5
DTI	Diffusion Tensor Imaging
EEG	Electroencephalography
fMRI	Functional Magnetic Resonance Imaging
GABA	Gamma-Aminobutyric Acid
GWAS	Genome-Wide Association Study
HTR1B	Serotonin Receptor 1B
IGD	Internet Gaming Disorder
KLF	Kruppel-Like Factors
LORETA	Low-Resolution Brain Electromagnetic Tomography
MRI	Magnetic Resonance Imaging
N/A	Not Applicable
PET	Positron Emission Tomography
QEEG	Quantitative Electroencephalography
RCT	Randomized Controlled Trial
SCL	Skin Conductance Level
SN	Salience Network
SMR	Sensorimotor Rhythm
TBR	Theta/Beta Ratio
TPN	Task-Positive Networks
